# Utility of a 40-minute LH level after depot leuprolide for diagnosis and treatment monitoring in girls with CPP

**DOI:** 10.1007/s40618-026-02925-0

**Published:** 2026-05-26

**Authors:** Didem Helvacioglu, Nilsu Turkmen, Serap Turan, Tulay Guran, Belma Haliloglu, Busra Gurpinar Tosun, Ahmet Kahveci, Abdullah Bereket

**Affiliations:** Department of Pediatric Endocrinology, Marmara University Faculty of Medicine, Prof. Dr. Asaf Ataseven Hospital, Istanbul, Türkiye

**Keywords:** Central precocious puberty, Diagnosis, Depot leuprolide, Luteinizing hormone (LH), GnRH stimulation test, Treatment monitoring

## Abstract

**Purpose:**

Gonadotropin-releasing-hormone (GnRH) stimulation test is used for assessment of gonadotropic activation in patients with central precocious puberty (CPP). However, it is invasive and GnRH preparation is not available in all countries. Depot-leuprolide acetate (dLA), used for the treatment of CPP, stimulates gonadotropins briefly before suppressing them. This study aimed to evaluate the utility of luteinizing hormone (LH) level measured 40-minutes after dLA injection for assessing gonadotropic activation compared to GnRH stimulation test.

**Methods:**

In this prospective study, girls with idiopathic CPP were randomized to receive dLA 3.75 mg/4 weeks IM, 11.25 mg/12 weeks SC or 11.25 mg/12 weeks IM. A standard GnRH test was performed before (baseline) and after 6 months of treatment. Forty minutes after the first dLA injection, LH and follicle-stimulating hormone (FSH) levels were measured, and the same measurements were repeated after 6 months of treatment.

**Results:**

The study recruited 132 girls. Peak LH on the GnRH test did not differ from the 40-minute-post-dLA LH levels [11.0(6.8–17.1) vs. 9.4(5.8–16.6); *p* = 0.06]. There were no significant differences between the treatment groups. Using the GnRH test peak of LH ≥ 5 IU/L as the gold-standard, ROC analysis identified the optimal cut-off of post-dLA injection LH ≥ 5.4 IU/L on initial measurement (sensitivity = 86%, specificity = 90% and AUC = 0.91). At 6 months, peak LH levels in treatment groups were similarly suppressed (2.1 ± 1.6, 2.5 ± 1.8, 2.2 ± 0.9; *p* = 0.2011). Furthermore, post- dLA LH cut-off of < 3.07 IU/L yielded an AUC = 0.83, sensitivity = 80.8% and specificity = 75.5%.

**Conclusion:**

The 40-minute post-dLA LH provided clinically useful information concerning gonadotropic activity for both diagnosis and treatment monitoring and may provide a practical and clinically informative alternative in selected settings, particularly when standard GnRH testing is not readily available.

**Supplementary Information:**

The online version contains supplementary material available at 10.1007/s40618-026-02925-0.

## Introduction

Central precocious puberty (CPP) in girls is defined as the onset of secondary sexual characteristics in a progressive manner before the age of 8 years due to early activation of hypothalamic-pituitary-gonadal (HPG) axis. The gonadotropin-releasing hormone (GnRH) stimulation test is the gold standard to investigate premature activation of the HPG axis in cases with clinical and anthropometric signs and symptoms of early puberty [1]. The GnRH test is also used to assess the efficacy of treatment, the clinical aim of which is gonadotropin suppression, during treatment of CPP with long-acting GnRH analogues [2]. The GnRH stimulation test requires an intravenous (iv) cannula insertion, GnRH injection and multiple blood samples at different time points, generally 3–5 separate samples. The test can be burdensome, especially in young children, as it requires multiple blood sampling through the iv cannula, is often painful and costly. In addition, a commercial preparation of synthetic GnRH is not always available, making the applicability of the test difficult at times.

In recent years, in countries where GnRH preparation is not readily available, the use of LH levels measured after short-acting gonadotropin-stimulating hormone analogue (GnRHa) injection has emerged as an alternative to the GnRH stimulation test [3–5]. However, studies with long-acting GnRHas are limited. In 2002, Bhatia et al. [6] showed that after injection of long-acting depot Leuprolide, serum LH levels peaked between 15 and 30 min after the injection and plateaued between 45 and 120 min. A limitation of this study was that the data obtained after depot Leuprolide injection were not compared with the results of the GnRH test, the gold standard in the diagnosis of CPP. In 2004, Brito et al. [7] compared the LH levels taken at 120 min after Leuprolide injection with the LH levels in the standard iv GnRH stimulation test in 18 girls with CPP using long-acting depot Leuprolide for evaluation of treatment efficacy. There is no published study on whether long-acting (depot) GnRH analogues used in the treatment of CPP can also be used for the diagnosis of CPP. Long-acting GnRH analogues, such as depot Leuprolide acetate (dLA), have been used as standard therapy in CPP for about 50 years [8]. These drugs act through desensitization and down-regulation of GnRH receptors in pituitary gonadotropic cells. Thus, gonadotropin release is gradually inhibited after the initial excitatory agonist phase [9, 10]. In the formulation of dLA, Leuprolide is enclosed in a capsule consisting of glycolic and lactic acid copolymers, facilitating slow release over around 28 days. The preparation also includes some free Leuprolide and this circulates systemically within minutes after injection. This free leuprolide stimulates gonadotropins during the first few hours, as it essentially behaves as a fast-acting GnRH analogue. [9, 10]. Taking advantage of this effect, our primary aim was to test the diagnostic usability of measuring the LH level in girls with CPP 40 min after injection of the dLA. We also aimed to test the usability of 40 min-post- dLA injection LH level for monitoring treatment efficacy after six months of treatment with dLA.

Both monthly (3.75 mg) and quarterly (11.25 mg) forms of dLA are available and approved for the treatment of CPP. These preparations may be administered by either subcutaneous (SC) or intramuscular (IM) injection. Comparative studies on the effect of these two forms on post-injection gonadotropin levels are limited. Comparison of the effect of different dLA dose and modalities on gonadotropin levels after injection was one of our secondary aims. Finally, another secondary aim was to investigate whether there is any diagnostic loss when a single blood sample is taken (at 30–60 min) after standard iv GnRH stimulation test.

## Materials and methods

This prospective, randomized study included girls with idiopathic CPP who were evaluated, diagnosed, and treated at a tertiary pediatric endocrinology center (Department of Pediatric Endocrinology, Marmara University) between 2020 and 2024.

The criteria for the diagnosis of CPP were: (1) progressive breast development starting < 8 years of age associated with accelerated growth and skeletal maturation; and (2) a peak LH ≥ 5 IU/L on GnRH stimulation test [11]. All girls also had pelvic ultrasound evaluation. Uterine length > 34 mm, and ovarian volume > 2 cubic centimeters (cc) were taken as supportive criteria [11–13]. Ten patients who met all of these criteria with a basal LH > 0.3 IU/L but had LH peak of 2–5 IU/L on GnRH stimulation test were assumed to have CPP and also included [11–14].

Pubertal development was staged according to the Marshall and Tanner criteria [15]. Height and weight were measured by standard techniques, BMI was calculated using the standard formula (weight in kg/[height in m]^2^), and the respective standard deviation scores (SDS) were calculated based on local reference data [16–18]. Bone age was assessed by X-ray of the left hand, according to the method of Greulich and Pyle [19]. All patients had normal results on cranial magnetic resonance imaging (MRI). Patients with incomplete precocious puberty, peripheral precocious puberty or those who exhibited neurological symptoms or had central nervous system pathologies were excluded. The GnRH test was performed between 8:00 and 8:30 a.m. An intravenous cannula was inserted, and blood samples were collected for basal FSH and LH. Following intravenous administration of 2.5 µg/kg dose of GnRH, in the form of gonadorelin acetate (Ferring Pharmaceuticals Inc., Tarrytown, NY, USA), blood samples for FSH and LH were obtained at 30 and 60 min. FSH and LH were measured using chemiluminescent microparticle immunoassay on the ARCHITECH System (Beckman Coulter Diagnostics, CA, USA) according to the manufacturer’s protocol. The minimum detectable concentration was 0.07 IU/L for both FSH and LH.

Following the establishment of the diagnosis of CPP, patients were randomly assigned to one of three treatment regimens of depot leuprolide acetate (dLA) using sequential allocation. The families were not involved in the decision-making process regarding the type of medication or its frequency and route of administration. Investigators were unblinded, but laboratory technicians were blinded to treatment allocation. Allocation arms were:

Group 1: dLA 3.75 mg IM every 4 weeks; (*n* = 46)

Group 2: dLA 11.25 mg SC every 12 weeks; (*n* = 45),

Group 3: dLA 11.25 mg IM every 12 weeks. (*n* = 45)

Four participants in the 11.25 mg SC group discontinued the study before completion of follow-up and were excluded from the final analysis.

**Fig. 1:**
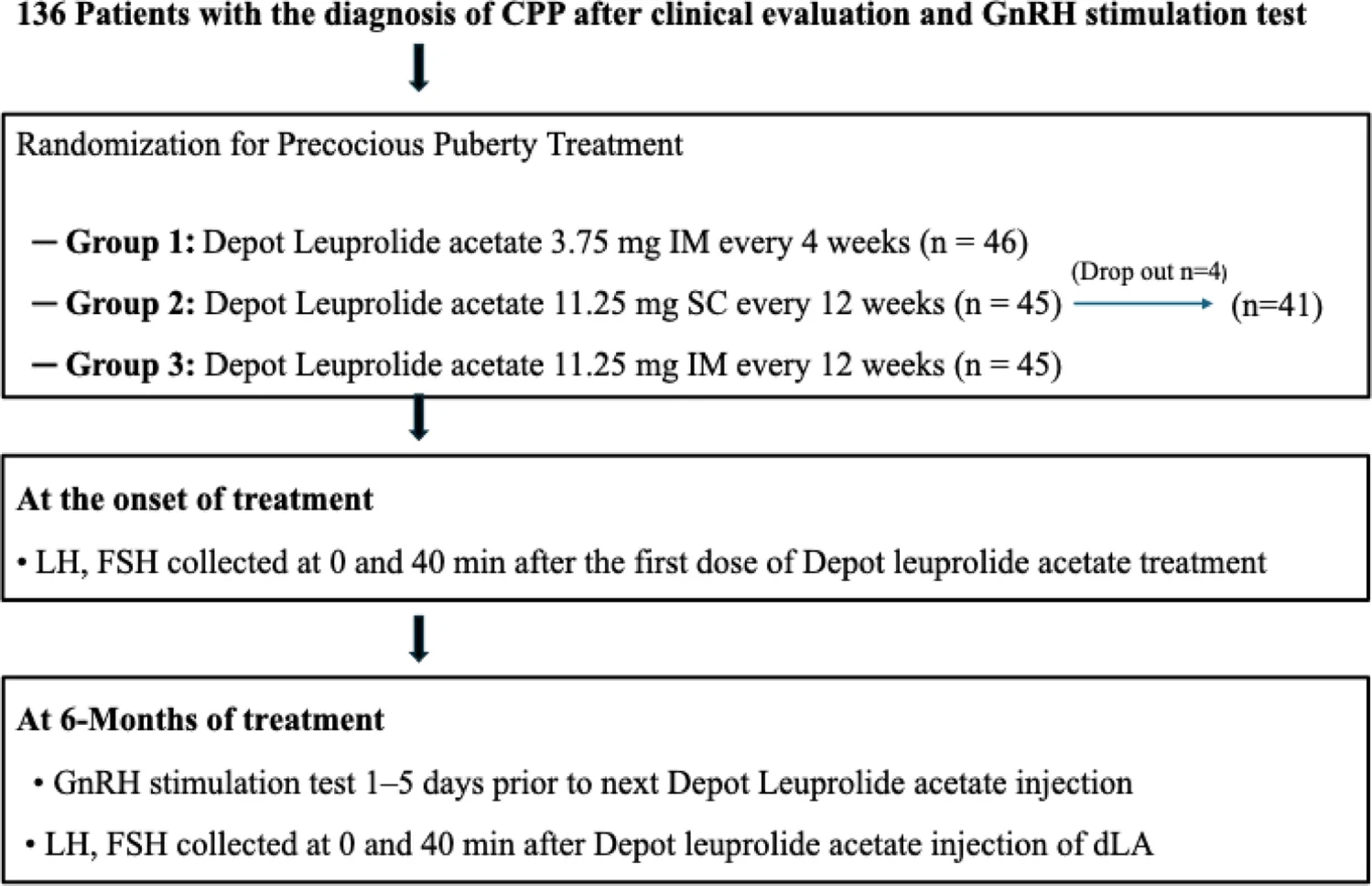
Study outline and schematic representation of the study design

All blood samples were collected in the morning (08:00–08:30 a.m.). At treatment initiation, serum LH and FSH were measured immediately before the first dLA injection (0 min) and at 40 min post-injection. At month 6, a standard GnRH stimulation test was performed in the morning 1–5 days prior to the scheduled dLA dose. At the day of scheduled subsequent dLA injection, LH and FSH were again measured immediately pre-injection (0 min) and at 40 min post-injection (Fig. 1).

### Statistical analysis

All statistical analyses were conducted using GraphPad Prism version 10 (GraphPad Software, San Diego, CA, USA). Continuous variables are reported as mean ± standard deviation (SD) or median with interquartile range (IQR), depending on distribution. Normality of distribution was assessed using the Shapiro–Wilk test. Comparisons among the three treatment groups were performed using one-way analysis of variance (ANOVA) for normally distributed continuous variables and the Kruskal–Wallis test for non-normally distributed variables. Categorical variables were compared using the chi-square test or Fisher’s exact test, as appropriate.

To assess the diagnostic performance of the 40-minute post-dLA LH level for pubertal activation, receiver operating characteristic (ROC) curve analysis was performed using the peak LH response on the GnRH stimulation test as the reference standard. For this analysis, a peak LH ≥ 5 IU/L was classified as pubertal activation. The area under the curve (AUC), sensitivity, specificity, optimal cut-off value, positive likelihood ratio, and negative likelihood ratio were calculated. The optimal cut-off was determined using the Youden index, which identifies the point maximizing the sum of sensitivity and specificity. 95% confidence intervals (95% CIs) were calculated for AUC, sensitivity, and specificity. To evaluate treatment efficacy at the 6-month follow-up, a second ROC analysis was performed using a peak LH < 3 IU/L on the GnRH stimulation test as the reference definition of adequate suppression [20–22], and the diagnostic performance of the 40-minute post-dLA LH level was assessed in the same way. Using the same GnRH-based reference standard, ROC analysis was also performed for pre-injection basal LH at 6 months. Correlations between GnRH-stimulated peak LH and LH values measured 40 min after dLA administration were evaluated at baseline and at 6 months, and at 6 months the correlation between GnRH-stimulated peak LH and pre-injection basal LH was also assessed, using Pearson or Spearman correlation analysis as appropriate according to data distribution. A two-sided p value < 0.05 was considered statistically significant.

## Results

A total of 136 girls were randomized to study. Four girls in Group 2 discontinued follow-up (did not want to continue with treatment n:2, moving to another city n:2). The final numbers of girls completed the study in each group were: Group 1 *n* = 46; Group 2 *n* = 41; and Group 3 *n* = 45. The mean ± SD chronological age across the whole study population was 8.1 ± 0.7 years and the mean bone age was 9.7 ± 1.2 years at diagnosis (Table 1). There were no differences in clinical and hormonal characteristics between the three treatment groups at the onset of therapy except a slightly lower bone age in Group 2.

**Table 1:** Clinical characteristics at treatment initiation and hormonal parameters at baseline and after 6 months of treatment in all patients

	All patients (*n* = 132)
Age (CA)	8.1 ± 0.7
Bone Age (BA)	9.7 ± 0.7
CA/BA	1.2 ± 0.9
Height SDS	0.9 ± 1.0
Weight SDS	1.0 ± 1.0
BMI SDS	0.8 ± 1.0
Baseline LH (IU/L)	0.7 (0.5–1.2)
Baseline FSH (IU/L)	3.2 (2.0–4.5.0.5)
Uterine length (mm)	34.8 ± 8.0
Uterine volume (cc)	2.6 (1.8–4.6)
Left ovarian volume (cc)	2.1 (1.3–3.3)
Right ovarian volume (cc)	2.0 (1.2–2.9)
Total ovarian volume	4.3 (2.7–6.0.7.0)
Basal LH (GnRH test)	0.7 (0.4–1.4)
LH at 30 min (GnRH test)	10.3 (6.5–17.3)
LH at 60 min (GnRH test)	9.2 (6.4–14.5)
Peak LH (GnRH test)	11.0 (6.8–17.1)
Basal FSH (GnRH test)	3.1 (2.2–4.8)
FSH at 30 min (GnRH test)	11.1 ± 4.0
FSH at 60 min (GnRH test)	13.0 ± 5.8
Peak FSH (GnRH test)	13.1 ± 5.8
Peak LH/FSH (GnRH test)	0.9 (0.6–1.6)
40-min LH (1st dLA)	9.4 (5.8–16.6)
40-min FSH (1st dLA)	10.8 ± 4.6
40-min LH/FSH (1st dLA)	1.0 (0.6–1.6)
Peak LH (6-month GnRH test)	2.0 (1.4–2.8)
40-min LH (6-month dLA)	2.6 (1.9–3.4)

Results are presented as mean ± SD or median (IQR), according to data distribution

The median (25th–75th percentile) peak LH levels during the standard GnRH stimulation test were 10.8 (6.6–15.7), 10.1 (6.8–21.3) and 11.1 (7.0–16.9.0.9) IU/L for groups 1,2 and 3, respectively. Similarly, the LH levels measured 40 min after the initial dLA injection were 9.4 (6.6–17.2), 9.0 (5.5–16.4), and 9.5 (5.8–16.7) IU/L, with no statistically significant intergroup differences (Table 2). Among the 10 patients with peak LH < 5 IU/L at GnRH stimulation test, eight also had LH < 5 IU/L 40 min after dLA at the time of diagnosis. The individual responses to the GnRH stimulation test and 40-minute post-dLA injection at baseline and after six months of treatment are illustrated in Supplementary Fig. 1.

**Table 2:** Baseline clinical and hormonal characteristics of treatment groups at treatment initiation

	dLA 3.75 mg IM (*n* = 46)	dLA 11.25 mg SC (*n* = 41)	dLA 11.25 mg IM (*n* = 45)	*P*
Age (CA)	8.2 ± 0.6	7.9 ± 0.95	8.2 ± 0.5	0.06
Bone Age (BA)	9.8 ± 1.1	9.7 ± 1.3	9.9 ± 1.1	0.03
CA/BA	1.2 ± 0.1	1.2 ± 0.1	1.2 ± 0.1	0.66
Height SDS	0.9 ± 1.1	1.1 ± 1.0	0.8 ± 1.0	0.61
Weight SDS	1.0 ± 1.1	1.0 ± 1.0	1.0 ± 1.0	0.95
BMI SDS	0.8 ± 1.1	0.7 ± 1.0	0.8 ± 1.0	0.80
Baseline LH (IU/L)	0.8 (0.4–2.0.4.0)	0.6 (0.5–0.9)	0.8 (0.6–1.2)	0.22
Baseline FSH (IU/L)	3.4 (1.6–4.6)	2.7 (2.0–4.0)	3.7 (2.6–4.7)	0.06
Uterine length (mm)	35.0 (33.0–41.0)	32.0 (29.0–37.0)	35 (30.0–40.0)	0.10
Uterine volume (cc)	2.8 (1.6–4.6)	2.6 (1.9–4.2)	2.4 (1.6–4.7)	0.98
Left ovarian volume (cc)	2.3 (1.7–3.6)	1.7 (1.0–3.2.0.2)	2.3 (1.4–3.0.4.0)	0.57
Right ovarian volume (cc)	2.3 (1.4–3.1)	1.7 (1.0–2.6.0.6)	2.2 (1.5–3.1)	0.12
Basal LH (GnRH test)	0.9 (0.4–1.6)	0.7 (0.5–1.5)	0.7 (0.4–1.3)	0.61
LH at 30 min (GnRH test)	10.1 (6.3–16.0)	10.1 (6.4–21.3)	11.0 (6.9–16.9)	0.99
LH at 60 min (GnRH test)	9.5 (6.4–16.0)	9.1 (6.5–16.2)	9.3 (6.3–14.1)	0.97
Peak LH (GnRH test)	10.8 (6.6–15.7)	10.1 (6.8–21.3)	11.1 (7.0–16.9.0.9)	0.99
Basal FSH (GnRH test)	3.9 (2.4–5.5)	2.9 (2.0–4.1.0.1)	2.7 (2.0–4.2.0.2)	0.07
FSH at 30 min (GnRH test)	11.1 ± 4.7	11.4 ± 3.3	11.0 ± 3.8	0.61
FSH at 60 min (GnRH test)	12.5 ± 4.9	13.3 ± 3.7	13.3 ± 8.0	0.23
Peak FSH (GnRH test)	12.7 ± 4.8	13.4 ± 3.7	13.4 ± 8.0	0.27
30-min LH/FSH (GnRH test)	1.0 (0.7–1.8)	0.9 (0.6–1.6)	1.0 (0.7–1.7)	0.87
60-min LH/FSH (GnRH test)	0.8 (0.6–1.4)	0.8 (0.4–1.4)	0.8(0.5–1.3)	0.68
Peak LH/FSH (GnRH test)	0.9 (0.7–1.5)	0.8 (0.5–1.6)	1.0(0.5–1.6)	0.86
Basal LH (1st dLA)	1.0 (0.5–1.8)	0.8 (0.5–1.3)	0.7(0.5–1.9)	0.33
Basal FSH (1st dLA)	4.1 ± 2.5	3.1 ± 1.6	4.4 ± 3.4	0.07
Basal LH/FSH (1st dLA)	0.3 (0.2–0.5)	0.3 (0.2–0.5)	0.3(0.2–0.4)	0.86
40-min LH (1st dLA)	9.4 (6.6–17.2)	9.0 (5.5–16.4)	9.5(5.8–16.7)	0.66
40-min FSH (1st dLA)	11.1 ± 5.8	10.5 ± 3.9	10.8 ± 3.8	0.95
40-min LH/FSH (1st dLA)	1.1 (0.7–1.6)	1.0 (0.5–1.9)	1.0(0.6–1.4)	0.55

Results are presented as mean ± SD or median (IQR) according to data distribution

**Fig. 2:**
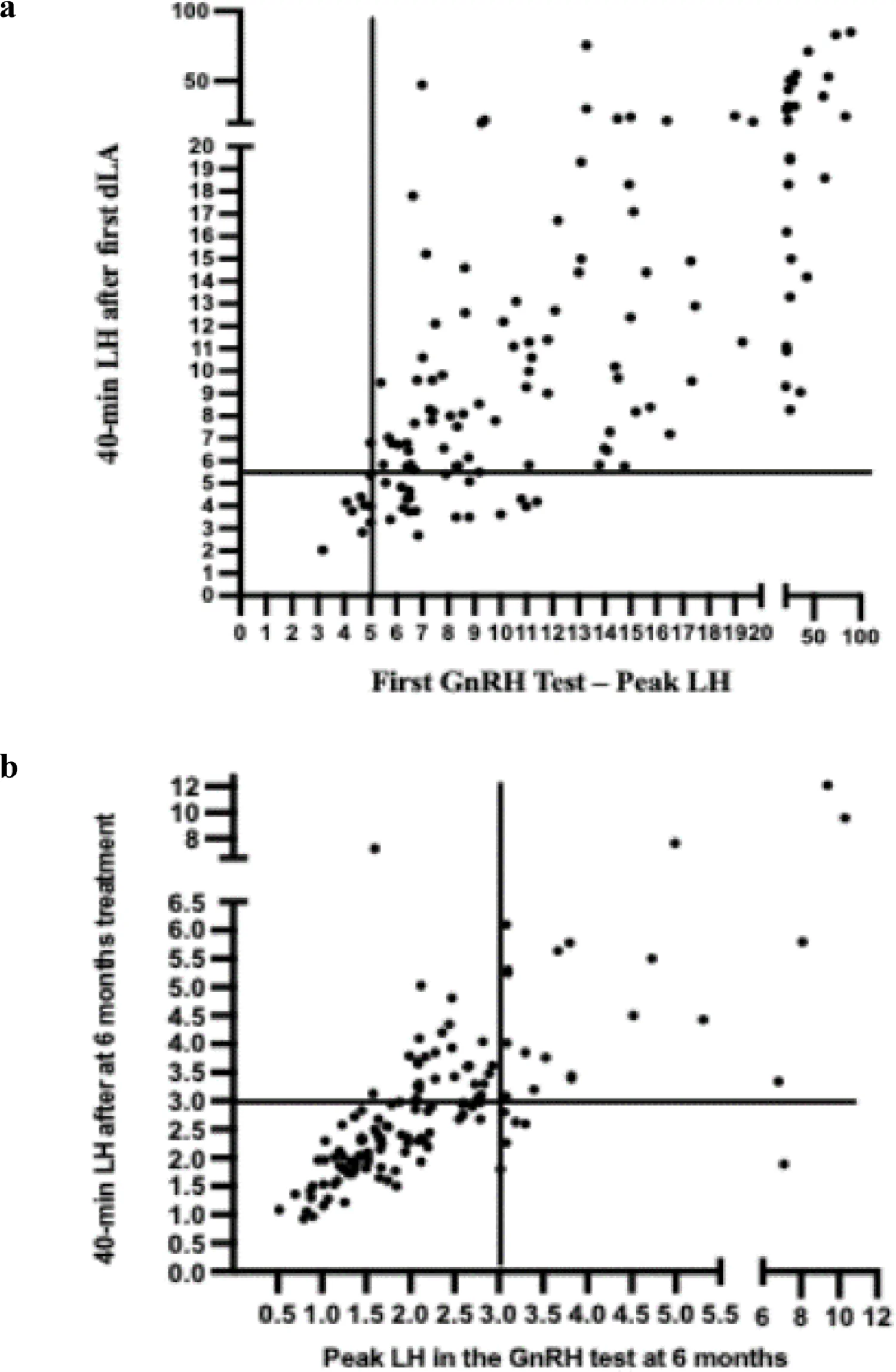
(**a**) Correlation between peak LH obtained during the GnRH stimulation test and the 40-minute post-dLA LH level at treatment initiation (**b**) Correlation between peak LH obtained during the GnRH stimulation test after 6 months of treatment and the 40-minute post-dLA LH level at the same time point

A strong positive correlation was observed between peak LH values obtained during the baseline intravenous GnRH stimulation test and the LH levels measured 40 min after the first dLA injection (Fig. 2a). This correlation was consistent across all three treatment groups and in the overall cohort of 132 patients (*r* = 0.71, *p* < 0.001). A majority (89%) of the patients whose peak LH > 5 IU/L on GnRH test at diagnosis, also had a 40-minute post-dLA LH > 5 IU/L.

**Fig. 3:**
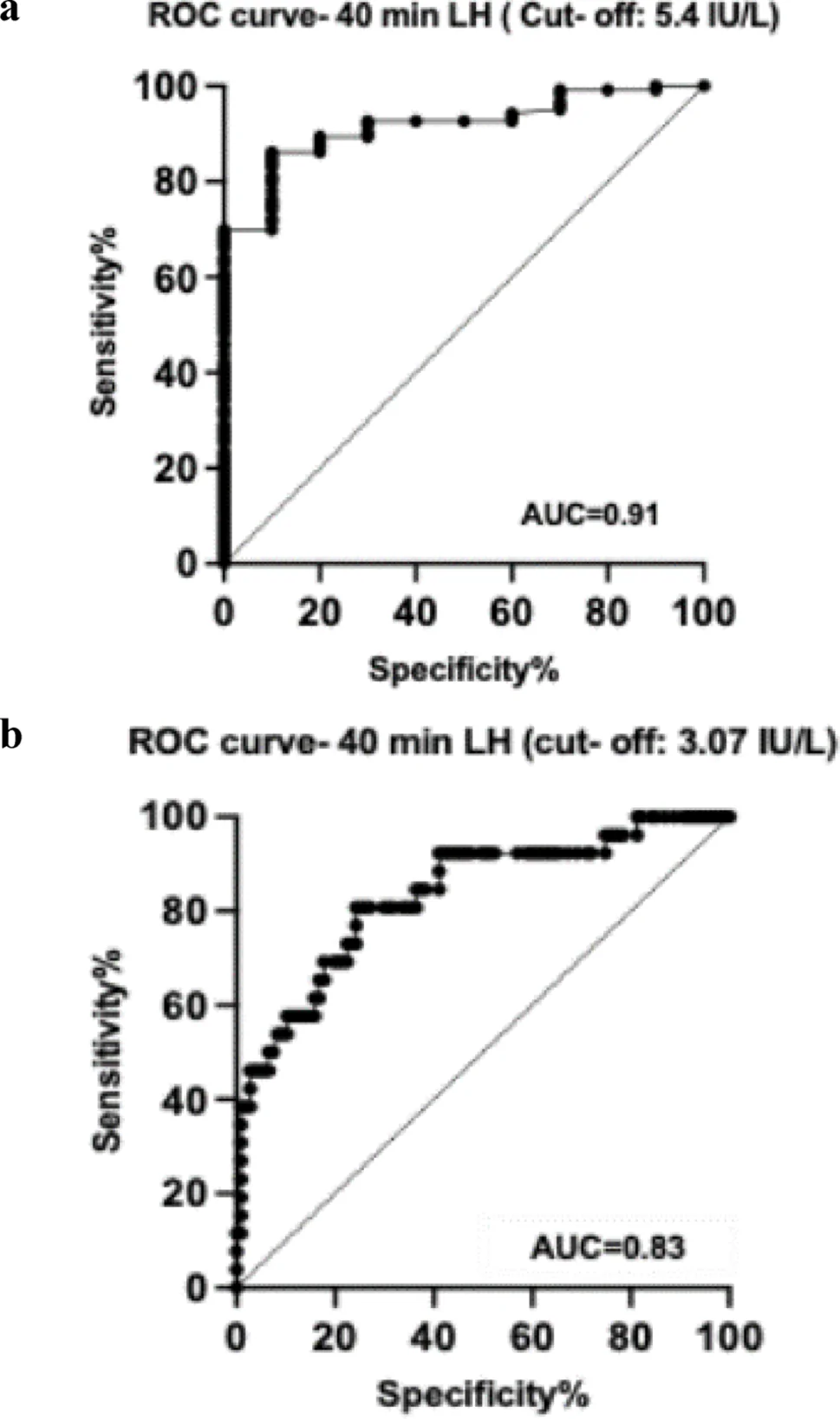
(**a**) Receiver operating characteristic (ROC) curve of the 40-minute post-dLA LH level for diagnosis of central precocious puberty using a GnRH-stimulated peak LH ≥ 5 IU/L as the reference standard (**b**) Receiver operating characteristic (ROC) curve of the 40-minute post-dLA LH level for treatment monitoring at 6 months using a GnRH-stimulated peak LH < 3 IU/L as the reference standard

Using the GnRH stimulation test peak LH ≥ 5 IU/L as the reference standard, ROC analysis identified a 40-minute post-dLA LH cut-off of 5.4 IU/L, with an AUC of 0.91 (95% CI, 0.844–0.974), sensitivity of 86.1% (95% CI, 78.8–91.1), specificity of 90.0% (95% CI, 59.6–98.2), a positive likelihood ratio of 8.61, and a negative likelihood ratio of 0.15 (Fig. 3a).

To assess treatment response (monitoring of gonadotropic suppression), a GnRH stimulation test was repeated at 6 months of treatment before the injection of next dLA. Peak LH values did not differ significantly between the groups (*p* = 0.20), with mean peak LH levels being similarly suppressed to 2.2 ± 1.6 vs. 2.6 ± 1.8 and 2.2 ± 0.9 IU/L in the GnRH test, and to 2.9 ± 1.9, 3.0 ± 1.4, and 2.9 ± 1.2 IU/L in 40-minute post-dLA injection for each group, respectively. A significant positive correlation was again observed between peak LH levels in the GnRH stimulation test and LH levels measured at 40 min after dLA injection after 6 months of treatment across all treatment groups (*r* = 0.74, *p* < 0.001) (Fig. 2b). Once again, most (88%) whose peak LH < 3 IU/L on GnRH test, also had a 40-minute post-dLA LH < 3 IU/L, confirming optimal suppression. For treatment monitoring, ROC analysis using a GnRH peak LH threshold of < 3 IU/L as the reference standard identified an optimal 40-minute post-dLA LH cut-off of 3.07 IU/L. The AUC was 0.83, with a sensitivity of 80.8% and specificity of 75.5%. The positive likelihood ratio was 3.32 and the negative likelihood ratio was 0.25 (Fig. 3b). Using the same GnRH-based reference standard, ROC analysis for pre-injection basal LH at 6 months identified an optimal cut-off of 0.76 IU/L, with an AUC of 0.71, sensitivity of 69.2%, and specificity of 69.5%, indicating only moderate discriminatory performance. Moreover, when both measurements were compared against GnRH-stimulated peak LH at 6 months, the correlation was stronger for 40-minute post-dLA LH than for pre-injection basal LH (*r* = 0.75, *p* < 0.001 vs. *r* = 0.56, *p* < 0.001).

Analysis of LH and FSH responses at 30 and 60 min after the first GnRH stimulation test demonstrated that mean LH at 30 min 10.3(6.5–17.3) IU/L was higher than that at 60 min 9.2 (6.4–14.5), *p* < 0.001, whereas mean FSH at 30 min (11.1 ± 4.0) IU/L was lower than that measured at 60 min (13.0 ± 5.8, *p* < 0.001). As a result, the average peak LH/FSH ratio was higher at 30 min than at 60 min (0.9 ± 0.4 vs. 0.7 ± 0.3, *p* < 0.001) (Table 1).

Furthermore, 105 of 132 subjects (79.5%) had their highest measured (peak) LH at 30 min whereas 27 of 132 subjects (20.5%) had the highest measured value at 60 min. Of the 122 subjects with peak LH level > 5 IU/L, 100 (82%) had LH > 5 IU/L at the 30 min measurement. Only two patients had LH < 5 IU/L at 30 min but LH > 5 IU/L at 60 min (Table 3).

**Table 3:** Frequency of peak LH at 30 and 60 min and cross-sectional and cumulative frequencies of LH ≥ 5 IU/L during the initial GnRH stimulation test

	30th min	60th min
Frequency of peak LH, *n*	105	27
Cross-sectional frequency of LH ≥ 5 IU/L n (%)	120 (90.9%)	117 (88.6%)
Cumulative frequency of LH ≥ 5 IU/L n (%)	120 (90.9%)	122 (92.4%)

## Discussion

In this prospective randomized study, we have demonstrated in 132 girls with idiopathic CPP that a single LH measurement following dLA injection correlated well with peak LH obtained in a standard GnRH stimulation test. Using the GnRH stimulation test (peak LH ≥ 5 IU/L) at diagnosis as the reference standard, ROC analysis identified an LH value of ≥ 5.4 IU/L measured at 40 min after the first dose of the dLA for diagnosing CPP. In addition, this approach proved to be useful for monitoring gonadotropic suppression (treatment efficacy) at six months following start of dLA treatment. In the present study, a GnRH-stimulated peak LH threshold of 3 < IU/L was used as the reference definition of adequate biochemical suppression during treatment, based on previous CPP treatment studies [20–22] in which similar stimulated LH thresholds were used as efficacy endpoints during GnRHa therapy. However, reported suppression cut-off values vary across studies according to the stimulation protocol, assay methodology, and sampling time [23–25]. Using this reference definition, a corresponding 40-minute post-dLA LH cut-off of ≤ 3.07 IU/L was identified by ROC analyses in the present study. The results were valid for all three treatment groups. Based on these results, we propose that a single LH measurement following injection of dLA may safely be utilized for both diagnosis and treatment efficacy in girls with CPP to simplify management, especially when GnRH ampul or facilities for testing are not readily available.

In recent years, in countries where GnRH preparation is not available, the use of LH level measured after short-acting GnRHa injection has been used instead of GnRH stimulation test. However, short-acting GnRHa is also not available in many countries. Studies using long-acting GnRHa for this purpose are scarce. In a pharmacokinetic study, after injection of long-acting dLA, serum LH levels peaked between 15 and 30 min after the injection and plateaued up to 120 min indicating that the free Leuprolide contained in long-acting depot Leuprolide is able to rapidly stimulate serum gonadotropin concentrations [6]. However, there was no comparison with the GnRH stimulation test in that study, which is considered the gold standard in the diagnosis of CPP. In another study, in 18 cases using long-acting depot Leuprolide for CPP, LH levels taken at 120 min after Leuprolide injection was compared with LH levels in the classical iv GnRH stimulation test for treatment monitoring. It was found that LH measurement after GnRH analogue is parallel to the classical iv GnRH test and can be used to monitor gonadotropin suppression in patients receiving CPP therapy [7]. In a similar study, Demirbilek et al. [26] demonstrated an LH value < 2.5 IU/mL 90 min after GnRHa injection was considered to be the cut-off for the determination of pubertal suppression (sensitivity and specificity was 100% and 88%, respectively) in girls with CPP during GnRHa treatment. The present study is the first to compare LH levels at GnRH stimulation test with LH levels measured 40 min after long-acting dLA for both diagnosis and treatment monitoring and in three different dLA dosage and modalities.

Considering evidence presented by Bhatia et al. [6], we chose 40 min for the measurement of gonadotropins after dLA injection as they suggested that a single serum sample for LH taken 30 to 60 min after injection of depot Leuprolide in children provided an appropriate and accurate assessment of treatment efficacy. In the present study, we further demonstrated that this approach can also be used for diagnosis of CPP. However, it should be noted that although the evoked LH response is the most important hormonal criterion, the diagnosis of CPP is based on a combination of clinical, anthropometric, radiological and hormonal criteria.Thus, it may be proposed that the patients who display clinical criteria for CPP could receive their first dose of dLA (3.75 mg) in the clinic and provide a post-injection blood sample for hormonal evaluation of CPP as quickly as 40 min afterwards. If the increase in LH 40 min after the dLA injection supports the diagnosis of CPP (> 5.4 IU/L), the diagnosis is confirmed and the patient can be advised to continue dLA treatment. If not, the patient can be followed further without treatment. Since post-dLA LH levels are compatible with post-GnRH stimulation LH levels, we believe that this proposed approach would significantly simplify the diagnostic process of CPP in terms of time, cost, and convenience, without sacrificing diagnostic accuracy. However, the use of a long-acting depot formulation (especially the 3-month formulation) leads to prolonged suppression of the hypothalamic-pituitary-gonadal axis, potentially exposing some patients in a diagnostic setting to unnecessary suppression.

In the present study, there were no significant differences among the three treatment groups regarding basal and GnRH stimulated gonadotropin levels or 40 min post-dLA gonadotropin levels, demonstrating similar stimulatory effect of all three treatment modalities. The same holds true for the evaluation of treatment efficacy, as the 40 min post-dLA gonadotropin concentrations also did not differ between the treatment groups after 6 months of treatment. For monitoring treatment efficacy, pre-injection basal LH showed only moderate discriminatory performance, whereas the 40-minute post-dLA LH measurement provided higher sensitivity and specificity. This is consistent with previous studies showing that basal or random unstimulated LH values may remain in the pubertal range despite otherwise adequate suppression during GnRHa therapy [27–29]. Thus, a 40-minute post-dLA LH measurement may be particularly useful when treatment efficacy needs to be assessed biochemically.

Although the present study is first, to the best of our knowledge, to compare the results of long-acting GnRHa with standard iv GnRH stimulation test for diagnosis of CPP, there are several studies comparing short acting GnRHas with a standard GnRH stimulation test. Eckert et al. [3] compared fast-acting, sc GnRHa (gonadorelin 100 µg) with standard iv GnRH stimulation test in 22 patients with CPP. They observed that the mean LH peak levels after iv GnRH and sc GnRH analogue administration were quite similar (*r* = 0.88). In a study with sc-administered, short-acting, aqueous Leuprolide 500 mg, Junquerira et al. [4] showed that peak LH value higher than 4.0 mIU/mL in the diagnosis of precocious puberty had the highest sensitivity (73%) and specificity (83.1%). Satashivam et al. [5] proposed that an LH value of 5 IU/L at 1–2 h after administration of short-acting Leuprolide acetate 20 µg/kg could be used in the diagnosis of CPP with a sensitivity of 78% and specificity of 100%. Thus, it appears that the combined sensitivity (86%) and specifity (90%) obtained in our study was even higher than those reported by studies using short acting GnRHas.

As a secondary aim, whether there was any diagnostic loss when a single blood sample was taken (at 30–60 min) during standard iv GnRH stimulation test was investigated. Average LH concentrations were higher and FSH concentrations were lower at 30 min compared to 60 min of the test, indicating that LH level peaks mostly at 30 min and slightly declines thereafter, whereas FSH reaches its peak at 60 min. This observation has important implications regarding diagnosis and monitoring of treatment in patients with CPP, since peak LH/FSH ratio, which is also a useful parameter in diagnosis of CPP [30], was significantly higher at 30 min than at 60 min. Thus, when comparing LH ratio in different studies it is important to know the timing of the blood samples used in this calculation. Our findings show that 82% of the patients would have been diagnosed with CPP, even if we had only taken the 30 min sample. Kandemir et al. [31] studied girls with CPP who underwent iv GnRH test with blood samples taken at 20, 40, 60 and 90 min. They found that 71% of the patients reached peak measured LH at 40 min and 98% of those with LH > 5 IU/L in their cohort had LH > 5 IU/l at 40 min [31]. These authors suggested that the GnRH stimulation test can be used for the diagnosis of CPP with high sensitivity (98%) and specificity (100%) when only a single sample was taken at 40 min. In combination with our findings, it appears that a single LH and FSH value measured at 30–40 min seems to be the most appropriate single time point to demonstrate the evoked LH value, to determine the LH/FSH ratio, and to be used for diagnosis.

A limitation of our study is that it was conducted in a clinically established CPP cohort rather than in an unselected diagnostic population. Therefore, some degree of selection bias cannot be excluded, and the generalizability of our findings to a purely diagnostic setting is limited.

In conclusion, in a large group of girls with CPP, it was demonstrated that a single 40 min LH level obtained after the first injection of long-acting GnRH analogue, dLA, was closely correlated with peak LH level obtained in a standard iv GnRH test with no difference across the three treatment groups. The same was true after six months of treatment for monitoring efficacy of the treatment eliminating the need to do an extra GnRH stimulation test. We propose that a 40 min-post dLA injection LH level provides ample information regarding gonadotropic activity and can be utilized both for diagnosis and treatment monitoring in CPP, especially when GnRH is unavailable.

## Supplementary Information

Supplementary Material 1.

## Data Availability

The datasets generated and analyzed during the current study are not publicly available due to patient confidentiality but are available from the corresponding author on reasonable request.
