# Supplementary material for: Utility of a 40-minute LH level after depot leuprolide for diagnosis and treatment monitoring in girls with CPP
Source: J Endocrinol Invest. 2026 May 26;49(9):2375–83. doi: 10.1007/s40618-026-02925-0 (PMC13498482; doi:10.1007/s40618-026-02925-0)
Supplement: Supplementary file 1 [file 40618_2026_2925_MOESM1_ESM.docx]

**Supplementary Fig. 1**
(a) Individual peak LH values during the GnRH stimulation test at baseline and after 6 months of treatment
(b) LH levels measured 40 minutes after depot leuprolide injection at baseline and after 6 months of treatment
